# Validation of the SOS-PD scale for assessment of pediatric delirium: a multicenter study

**DOI:** 10.1186/s13054-018-2238-z

**Published:** 2018-11-20

**Authors:** Erwin Ista, Babette van Beusekom, Joost van Rosmalen, Martin C. J. Kneyber, Joris Lemson, Arno Brouwers, Gwen C. Dieleman, Bram Dierckx, Matthijs de Hoog, Dick Tibboel, Monique van Dijk

**Affiliations:** 1 Intensive Care Unit, Departments of Pediatrics and Pediatric Surgery, Erasmus MC-Sophia Children’s Hospital, Office Sb-2704, P.O. Box 2060, 3000 CB Rotterdam, The Netherlands; 2 Department of Child and Adolescent Psychiatry and Psychology, Erasmus MC-Sophia Children’s Hospital, Rotterdam, The Netherlands; 3 Department of Biostatistics, Erasmus MC, Rotterdam, The Netherlands; 4 Department of Pediatrics, Division of Pediatric Critical Care Medicine, Beatrix Children’s Hospital, University Medical Center Groningen, University of Groningen, Groningen, The Netherlands; 5 Critical Care, Anaesthesiology, Peri – operative & Emergency medicine (CAPE), University of Groningen, Groningen, The Netherlands; 6 Department of Intensive Care, Radboud University Medical Center, Nijmegen, The Netherlands; 7 Department of Pediatrics, Division Pediatric Intensive Care, Maastricht University Medical Centre+, Maastricht, The Netherlands

**Keywords:** Pediatric delirium, Assessment tool, Iatrogenic withdrawal syndrome (IWS), Benzodiazepine, Sedation, PICU

## Abstract

**Backgrounds:**

Reports of increasing incidence rates of delirium in critically ill children are reason for concern. We evaluated the measurement properties of the pediatric delirium component (PD-scale) of the Sophia Observation Withdrawal Symptoms scale Pediatric Delirium scale (SOS-PD scale).

**Methods:**

In a multicenter prospective observational study in four Dutch pediatric ICUs (PICUs), patients aged ≥ 3 months and admitted for ≥ 48 h were assessed with the PD-scale thrice daily. Criterion validity was assessed: if the PD-scale score was ≥ 4, a child psychiatrist clinically assessed the presence or absence of PD according to the *Diagnostic and statistical manual of mental disorders* (DSM)-IV. In addition, the child psychiatrist assessed a randomly selected group to establish the false-negative rate. The construct validity was assessed by calculating the Pearson coefficient (r_p_) for correlation between the PD-scale and Cornell Assessment Pediatric Delirium (CAP-D) scores. Interrater reliability was determined by comparing paired nurse-researcher PD-scale assessments and calculating the intraclass correlation coefficient (ICC).

**Results:**

Four hundred eighty-five patients with a median age of 27.0 months (IQR 8–102) were included, of whom 48 patients were diagnosed with delirium by the child psychiatrist. The PD-scale had overall sensitivity of 92.3% and specificity of 96.5% compared to the psychiatrist diagnosis for a cutoff score ≥4 points. The r_p_ between the PD-scale and the CAP-D was 0.89 (CI 95%, 0.82–0.93; *p* < 0.001). The ICC of 75 paired nurse-researcher observations was 0.99 (95% CI, 0.98–0.99).

**Conclusions:**

The PD-scale has good reliability and validity for early screening of PD in critically ill children. It can be validly and reliably used by nurses to this aim.

**Electronic supplementary material:**

The online version of this article (10.1186/s13054-018-2238-z) contains supplementary material, which is available to authorized users.

## Background

Delirium is an acute dysfunction of the brain and is common in adult intensive care unit (ICU) patients with incidence up to 80% [1–3]. Reported prevalence rates of delirium in critically ill children range from 4% to 47% [4–10], and in a recent study even up to 56% in children below 2 years of age [9]. Delirious patients have disturbed consciousness and/or attention and other changes in cognition that develop over a short period of time. Their behavior may be described as hyperactive, hypoactive, or mixed [11]. In adults, delirium has been associated with, among other things, increased mortality risk, longer ICU stay, complications, and long-term cognitive deterioration [12–14]. In pediatric care, delirium is associated with prolonged stay and increased healthcare costs [15, 16]. Therefore, early identification of delirium in children is an urgent medical matter, as treatment of the underlying causes may minimize long-term consequences.

Three tools for assessing delirium in critically ill children have become available: the Cornell Assessment of Pediatric Delirium (CAP-D) [7, 10], the pediatric Confusion Assessment Method for ICU patients (pCAM-ICU) [8], and the preschool CAM-ICU (psCAM-ICU) [9]. Each has its own strengths and limitations.

Earlier we developed and validated the Sophia Observation Withdrawal Symptoms-scale (SOS) to assess iatrogenic withdrawal symptoms (IWS) in critically ill children aged 0–16 years [17, 18]. As IWS is a possible cause of delirium, symptoms of both conditions overlap [19, 20]. From a pilot study we concluded that the SOS scale, extended with a pediatric delirium (PD) component (SOS-PD scale), had promising validity and reliability [21]. We aimed to determine the measurement properties of the PD part of the SOS-PD scale for use by nurses, in terms of construct validity, criterion validity, and interrater reliability.

## Methods

### Design

A multicenter prospective observational study with repeated measures was conducted in four PICUs of university hospitals in the Netherlands. Data were collected over 6 months in each center, in the period from December 2015 through August 2016. Nurses applied the PD-scale (the PD component of the SOS-PD scale) at least three times a day, once every shift, in all eligible patients.

### Setting and study population

Children between 3 months and 18 years of age admitted to a PICU and with an expected length of stay of at least 48 h were eligible for inclusion. Exclusion criteria were the following: anticipated death within 48 h; neurological abnormality (e.g. severe psychomotor delay, encephalitis); comatose, or deeply sedated (COMFORT behavior score < 11, or Richmond Agitation-Sedation Scale (RASS) < −3), or paralysis through neuromuscular blocking agents during the whole admission period, which would make behavioral assessment impossible. The local institutional review board (EMC-2013-545) approved the study and patients were included after informed consent from the parents.

### Instruments

**Fig. 1:**
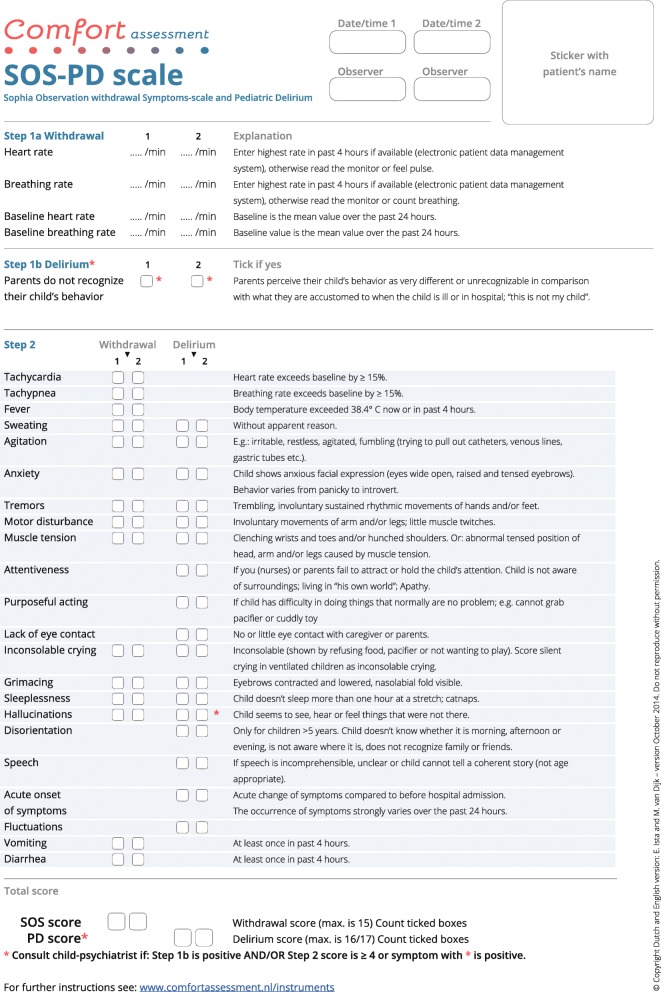
Sophia Observation Withdrawal Symptoms Scale (SOS-PD)

The PD-scale consists of 17 items that represent symptoms of PD and an item that reflects the perspective of the child’s parents (“parents do not recognize their child’s behavior as normal”) (Fig. 1) [21]. Presence of a symptom is scored as “yes” if it was observed at any moment during the previous 4 h. If the items “hallucinations” or “parents do not recognize their child’s behavior” were positive, a score of 4 points was assigned. Therefore, if only the item hallucinations is scored as yes, it overrides the PD score < 4 – by which the threshold for the index test (SOS-PD scale) is reached. The same holds for the parents’ item. The maximum sum score of the PD-scale is 17 points. In our pilot study, the sensitivity was 96.8% (95% CI, 80.4–99.5%) and the specificity was 92.0% (95% CI, 59.7–98.9%) for the cutoff point ≥4 [21]. The intraclass correlation coefficient (ICC) from 16 paired nurse-researcher observations was 0.90 (95% CI, 82.7–99.4) [21]. Given the lack of data on false-negatives and the small sample size of the pilot study, the validity should be reproduced in a large multicenter study.

The construct validity was tested by comparing outcomes on the PD-scale with outcomes on the revised CAP-D [10]. The CAP-D is a reliable and valid tool for assessing delirium in critically ill children of 0–18 years of age and consists of eight items scored 0–4. A total score ≥9 is consistent with a diagnosis of delirium [10].

### Study procedure

Local research nurses who had been trained by the principal investigator (EI) trained all nurses involved in the study in applying the PD-scale. The research nurses were also trained in applying the CAP-D. After the unit nurses had received theoretical instruction, they applied the PD-scale while watching video material of three cases of PD diagnosed by a child psychiatrist (one case of hypoactive delirium and two cases of hyperactive delirium). The nurses’ scores were compared with the reference score provided by the instructor. Discrepancies between the reference score and nurses’ scores were explained and advice was given for observation in clinical practice.

Patients meeting the inclusion criteria were included in the study 48 h after admission. Assessments were not performed if the patient was over sedated (COMFORT behavior score <11 or RASS < −3) or comatose [10, 22, 23]. In this situation, clinical assessment for delirium is useless. Caregiving nurses applied the PD-scale every shift at set times, i.e. 0400, 1400, and 2000 h.

A PD-scale score ≥4 or a positive score on the item “hallucination” was reason to consult a child psychiatrist (“suspected delirium”, Additional file 1: File S1). This threshold was based on our previous study and defined prior to the data collection [21]. If the patient was still admitted the next day, PD-scale assessments and psychiatrist consultation were repeated.

To estimate the number of false negatives, a random selection of patients with a PD-scale score <4 was assessed by a child psychiatrist on weekdays (Additional file 1: File S1). If at a particular moment more than one patient had been assigned a SOS-PD score <4 (random sample) the the local study coordinator determined, with the use of opaque envelops containing the bed numbers of the eligble patients, which patient (with a maximum of 2 patients) would undergo psychiatric evaluation. The psychiatric standardized examination is non-invasive (no physical examination) and includes a succinct assessment of consciousness, attention, orientation, or other disturbances in cognitive functioning according to the DSM-IV criteria (“gold standar”) [11]. The psychiatrist requested additional information (allo-anamnesis) from the parents, treating physician, and nurse, and reviewed the medical history (medication use, type of illness, etc.). The full examination took 40–50 min. The attending nurse performed the PD assessment within 1 h before the psychiatric evaluation and did not discuss the results with the child psychiatrist. A diagnosis of delirium was reported to the medical team so that appropriate measures could be taken. Regarding the first item on the PD-scale, the nurses asked the parents whether their child behaved differently from “normal” in relation to illness and previous days (e.g. hallucinations, withdrawn, making no eye contact). If parents said “This now is not my child, I don’t recognize my child anymore”, this could be a sign of the hyperactive as well as the hypoactive form of delirium, especially in very young children.

In addition, to establish the interrater reliability in clinical (bedside) circumstances a research nurse in each center performed at least 15 paired observations, a convenience sample, with a caregiving nurse, based on the availability of patients and nurses. Further, during weekdays the research nurse independently assessed patients with the CAP-D while simultaneously the attending nurse was performing the PD assessment.

#### Additional clinical data

The following additional data were collected: birth date, gender, reason for admission, type of respiratory support, length of stay in the PICU, Pediatric Risk of Mortality Score (PRISM) III-24 (first 24 h), type of continuous infusion of sedatives and opioids, and significant clinical developmental delay, based on clinical assessment and/or parental report of developmental problems that affected the child’s behavior or ability to communicate. Children with mild or transient history of developmental problems (i.e., needing occupational therapy for motor or speech delays) but who currently did not have communication or behavior problems were classified as normal [10].

#### Sample size calculation

Assuming a delirium prevalence of 15%, and point estimate for sensitivity of 90%, a sample size of 22 patients with delirium was required for the analysis. The sample size was calculated to ensure the appropriate number of patients necessary to provide the lower bound for the 95% CI of 70%. With 126 patients without delirium, the lower bound of the 95% CI for specificity was estimated to be 96%. So, a sample size of 148 patients was required for the analysis. We expected that 10% of patients would have missing data. Thus, the total sample size required for the investigation was 163 patients. This corresponded with 45 patients per participating PICU (*n* = 4).

### Statistical analysis

Demographics were summarized using descriptive statistics. The frequencies of the PD-scale items were divided into 3e groups: (1) assessments in the whole patient group; (2) assessments in delirious patients after diagnosis by the child psychiatrist; and (3) assessments in delirious patients till 48 h after diagnosis by the child psychiatrist. Data on patients with and without delirium were compared using the chi-square test for categorical variables and the Mann-Whitney test for continuous variables. These tests were also used to compare the demographic variables (age, reasons for admission, respiratory support, and severity of illness) of patients with suspected delirium and random psychiatric assessment.

#### Reliability

Interrater reliability of the PD-scale was assessed using Cohen’s kappa for the dichotomous items and using an intraclass correlation coefficient (ICC) for average measures with a two-way random effects model for continuous data [24]. Cohen’s kappa > 0.65 was considered satisfactory [25].

#### Validity

Criterion validity was defined as the ability of the PD-scale to classify patients into normal and PD categories compared with the psychiatrist’s verdict using the DSM-IV criteria. This was assessed by calculating the sensitivity, specificity, positive and negative predictive value, and the positive and negative likelihood ratios. Because the child psychiatrist was consulted for only a random selection of patients with a PD score <4, and in all patients with a PD score ≥4, naive estimates of test characteristics will be biased due to partial verification bias. To correct for this problem, we applied the formulas of Begg et al. [26] with the PD score as an ordinal variable. A receiver-operating-characteristic curve and associated area under the curve with adjustment for partial verification bias were calculated using the method of Zhou [27]. For this analysis one observation for each patient was used, that is the first assessment by the psychiatrist for patients with at least one such assessment, and the first observation of the PD-score for all patients without an assessment by the psychiatrist.

We tested the construct validity of the PD-scale by comparing the scores with the CAPD scale scores, on the assumption that both measure delirium in critically ill children. In line with the Consensus-based standards for the selection of health measurement instruments (COSMIN) checklist, we hypothesized that the correlation coefficient between the CAP-D and PD-scale was moderate and at least 0.65 [28, 29].

## Results

**Fig. 2:**
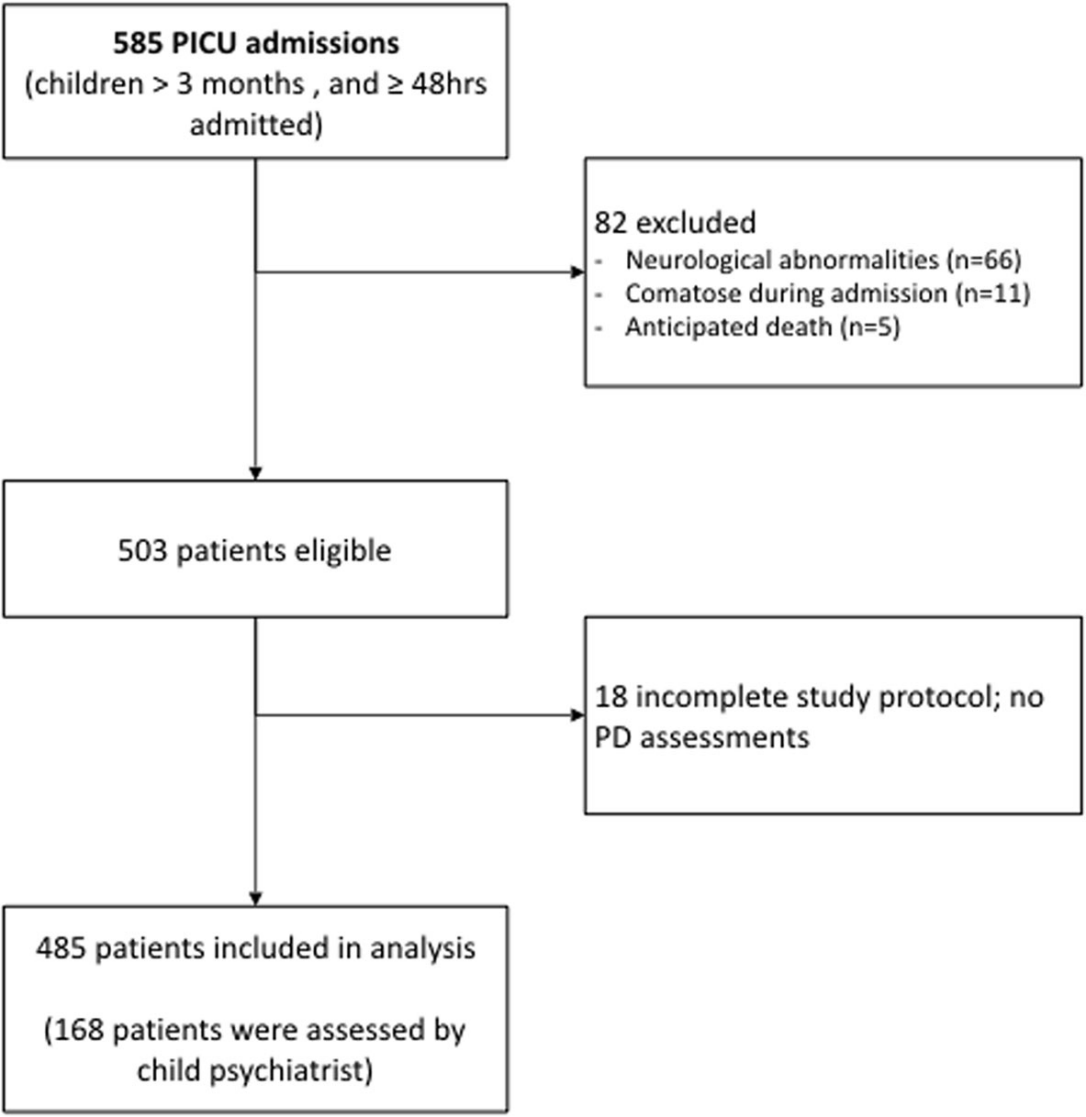
Inclusion flowchart. PICU, pediatric intensive care unit

Of 585 patients screened for eligibility, 100 were excluded (see Fig. 2). The 485 patients included in the analysis had a median age of 27.0 months (IQR 8–102) and 59.6% of them were boys. Almost half (42.5%) were admitted with respiratory failure and 56.9% (276/485) spent time on the ventilator (Table 1). More than half of the patients received sedatives (e.g. midazolam, lorazepam) or opioids, 56.1% (272/485) and 57.3% (278/485), respectively (Table 2).

**Table 1:** Demographic variables for the patient groups with and without confirmed delirium (*n* = 485)

Characteristic	Patients without confirmed delirium (*n* = 437) Number (%)	Patients with confirmed delirium (*n* = 48) Number (%)	*P* value
Gender			
- Female	179 (40.9)	17 (35.4)	0.536
- Male	258 (59.1)	31 (64.6)	
Age (months)*	25 (8–100)	39.5 (16–125)	0.042
Age categories			
- 3–24 months	215 (49.2)	19 (39.6)	0.540
- 2–5 years	70 (16.0)	8 (16.7)	
- 5–12 years	89 (20.4)	11 (22.9)	
- > 12 years	63 (14.4)	10 (20.8)	
Reason for admission:			
- Respiratory failure	186 (42.6)	20 (41.7)	0.152
- Cardiac (including cardiac surgery)	70 (16.0)	2 (4.2)	
- Postoperative (elective)	72 (16.5)	6 (12.5)	
- Infections	32 (7.3)	5 (10.4)	
- Trauma	26 (5.9)	4 (8.3)	
- Neurology	28 (6.4)	5 (10.4)	
- Others	23 (5.3)	6 (12.5)	
Type of respiratory support			
- None	61 (14.0)	1 (2.1)	<0.001
- Oxygen	129 (29.5)	2 (4.2)	
- Non-invasive ventilation	15 (3.4)	1 (2.1)	
- Ventilated (conventional)	218 (49.9)	40 (83.3)	
- HFO-ventilation	14 (3.2)	4 (8.3)	
Length of stay ICU (days)*	6 (4–10)	12 (7–20)	<0.001
Severity of illness - PRISM III*	5 (1.0–9.5)	6 (1.0–12.0)	0.182
Died during ICU stay	11 (2.5)	1 (2.2)	1.000
Developmental delay	40 (9.2)	2 (4.2)	0.413

*PRISM* Pediatric Risk of Mortality, *HFO* high frequaency oscillation

*Median (IQR)

**Table 2:** Continuous infusion of sedatives and opioids (*n* = 485)

Continuous infusion of sedatives and opioids administered	Patients without confirmed delirium (*n* = 437) Number (%)	Patients with confirmed delirium (*n* = 48) Number (%)	*P* value
Sedatives received			
- Benzodiazepines (midazolam, lorazepam)	226 (51.7)	46 (95.8)	<0.001
Opioids received			
- Morphine	233 (53.3)	45 (93.8)	<0.001
Number of different sedative classes received^a^			
- 0	178 (41.0)	2 (4.2)	<0.001
- 1	54 (12.5)	1 (2.1)	
- 2	129 (29.7)	22 (45.8)	
- 3 or more	75 (16.8)	23 (47.9)	
Median (IQR)	1 (0–2)	2 (2–4)	<0.001

*Median (IQR)

^a^Different sedative classes include opioids, benzodiazepines, α2-adrenergic agonists, propofol, barbiturates, ketamine, and chloral hydrate

In total, 48 patients (9.9%) were diagnosed with delirium. One patient was identified as delirious twice during the PICU admission (Table 3). These patients were admitted for significantly longer (*p* < 0.001), were significantly older (*p* < 0.042) and significantly more of them spent time on the ventilator (*p* < 0.001) than patients without delirium. Overall, 182 psychiatric evaluations were performed in 168 patients, 62 evaluations for suspected delirium and 120 evaluations for at-random assessment (Table 3). Informed consent was provided for 168 of the 173 patients (97%) who were eligible for psychiatric evaluation. No data were excluded in the case of inconclusive or uninterpretable index or reference tests. The time interval between the SOS-PD assessment and psychiatrist’s assessment was less than 1 h in more than 95% of cases. These two groups of patients were not significantly different with respect to demographic and clinical variables.

**Table 3:** Total numbers of psychiatric assessments performed

	Psychiatrist: delirium +	Psychiatrist: delirium -	Total
PD scale ≥ 4	48	14	62
PD scale < 4	1	119	120
Total	49	133	182

*PD* pediatric delirium

A total of 49 positive psychiatric assessments had been performed in 48 patients with suspected delirium. In total 48 patients were diagnosed as delirious. One patient was identified as delirious twice during the pediatric ICU admission

### PD-scale scores

**Fig. 3:**
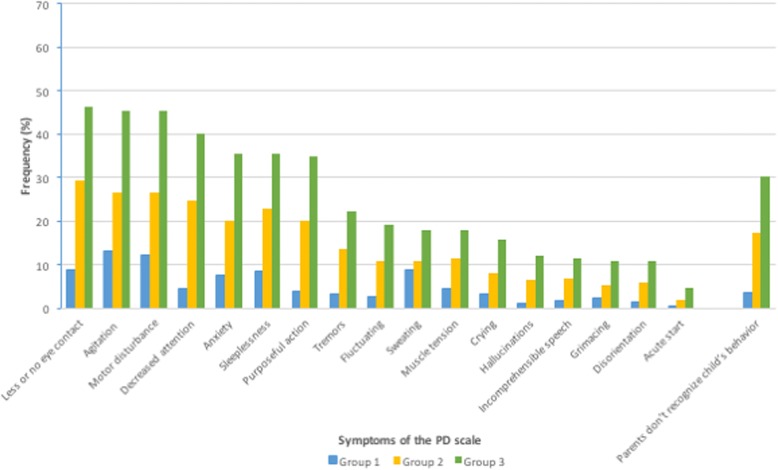
Observed delirium symptoms of the Pediatric Delirium Scale (PD scale). Every item is represented by 3 bars. The blue bars (Group 1) show the frequency of the item for all assessments (5207) in the whole patient group (*n* = 485). The yellow bars (Group 2) show the frequency of the item for assessments (758) in delirious patients after diagnosis by the child psychiatrist (*n* = 48). The green bars (Group 3) show the frequency of the item for assessments (229) in delirious patients up to 48 h after diagnosis by the child psychiatrist (*n* = 48)

Nurses performed 5207 PD-scale assessments in 485 patients, which corresponds to a median number of 5 (IQR 2–13) assessments per patient. The median PD score was 0 (IQR 0–1; *n* = 5207) for the total population, and the median number of days that patients were assessed was 3 days (IQR 1–7). The frequencies of items of the PD-scale are shown in Fig. 3 for the different groups. Purposeful acting, agitation, anxiety, motor disturbance, attentiveness, lack of eye contact, and sleeplessness (34.9–46.3%) were the most prevalent in patients with delirium.

### Measurement properties of the PD-scale

#### Reliability

The attending nurse and the researcher made 75 paired PD-scale assessments. The ICC for the total sum score between the attending nurse and researcher was 0.99 (95% CI, 0.98–0.99). The interrater reliability (Cohen’s kappa) for the individual items ranged from 0.79 to 1.0 (See Additional file 1: File S2).

#### Criterion validity

**Fig. 4:**
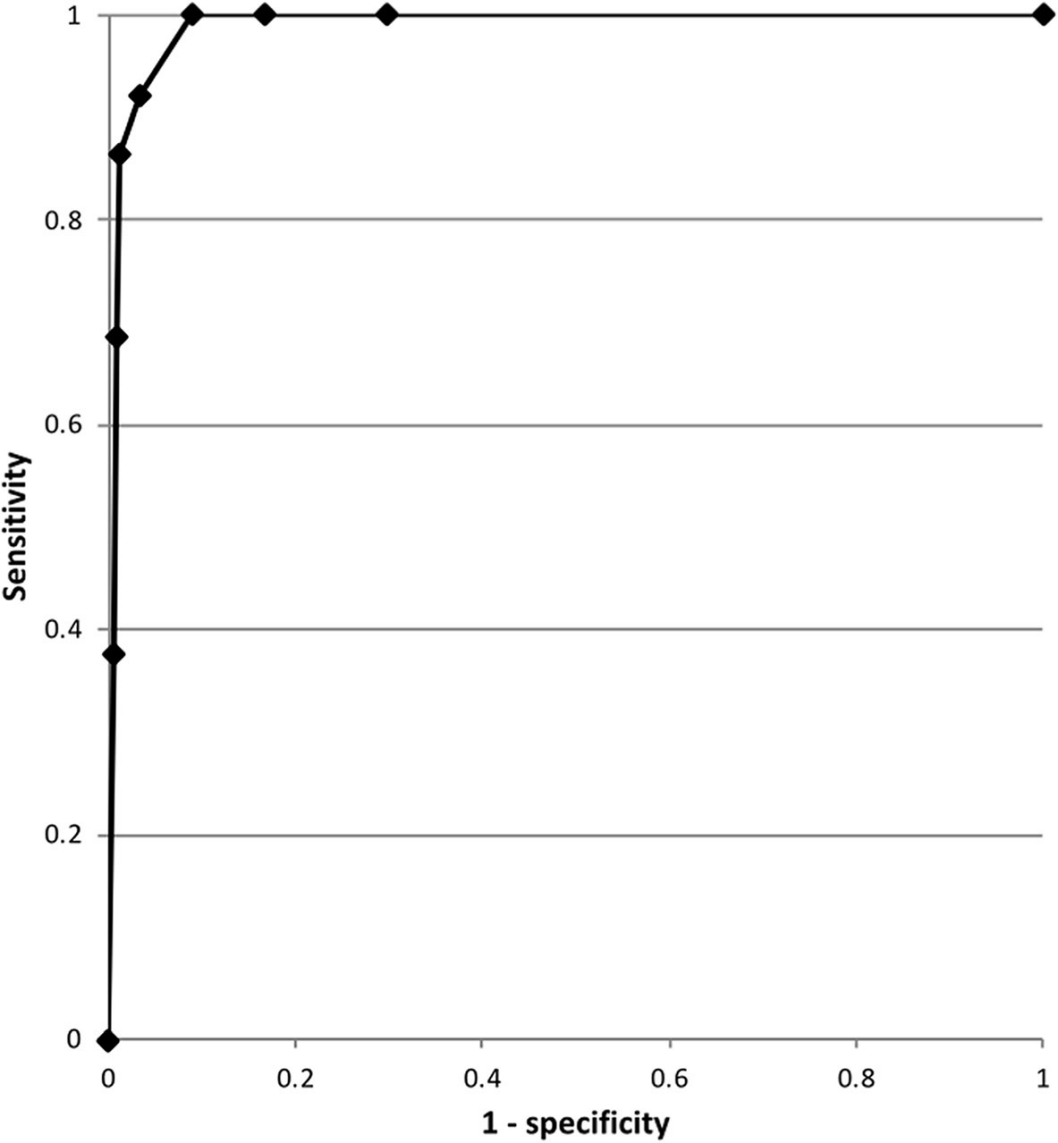
Receiver-operating characteristic (ROC) curve for performance of the Pediatric Delirium (PD) part of the Sophia Observation Withdrawal Symptoms Scale (SOS-PD). The area under the cure is 0.989. The calculation of the ROC curve has been adjusted for partial verification bias using the method of Zhou [27]

To establish the criterion validity of the PD-scale, the first child psychiatrist assessment was compared with the PD score in the randomly selected patients (PD-scale score <4; *n* = 114) and for the patients with suspected delirium (PD-scale score ≥4; *n* = 54), respectively (Table 4). In three cases in which the PD score was ≥ 4, the child psychiatrist was not consulted for logistic reasons. The sensitivity was 92.3% and the specificity was 96.5% after correction for verification bias, for a PD score ≥4. Figure 4 shows the receiver-operating characteristic-curve, in which the area under the curve was 0.989. The positive predictive and the negative predictive values (without adjustment for verification bias) were 76.4% and 99.1%, respectively. The positive likelihood ratio and the negative likelihood ratio (based on partial verification bias) were 26.5 and 0.08, respectively. Delirium was diagnosed on median day 8 (IQR 5–14) after admission. The estimated prevalence of PD was 10% (with adjustment for verification bias). The observed prevalence of delirium (without adjustment for verification bias) was highest in patients who had been on invasive ventilator support at any time (44/232, 18.9%) followed by the age group > 12 years of age (10/63, 15.9%). The hyperactive subtype of delirium was the most common, affecting 43.8% of patients. The hypoactive subtype occurred in 33.3% and the mixed-subtype in 22.9% of patients. At a cutoff point ≥4, there were one false-negative PD assessment and 14 false-positive screens among the observations with an assessment by the child psychiatrist. The false negative PD assessment was a 16-month-old boy with an infection who was on non-invasive ventilation. The caregiving nurse assessed the child and scored 3 points on the PD-scale (agitation, purposeful acting, and inconsolable crying). The child psychiatrist reported signs of decreased awareness and attention, cognition, withdrawn affection, and fluctuation, and concluded the patient had hypoactive delirium. In the next shift the score on the PD-scale was 6.

**Table 4:** Numbers of psychiatric assessments performed for criterion validity – correcting for verification bias

	Psychiatrist: delirium +	Psychiatrist: delirium -	No evaluation	Total
PD scale ≥ 4	41	13	3	57
PD scale < 4	1	113	314	428
Total	42	126	317	485

Based on correction for verification bias, only the first psychiatric assessment and the corresponding pediatric delirium (PD)-assessment were evaluated; 42 patients were diagnosed with delirium. In comparison to Table 3, the first psychiatric assessment in 7 patients was performed in the framework of random assessment and they were not diagnosed as delirious. During the pediatric ICU admission a second psychiatric assessment was performed in the case of suspected delirium. These assessments were excluded in the analysis for correcting for verification bias

#### Construct validity

In 144 cases, we compared the PD-scale scores of the caregiving nurse with the CAP-D scores of the research nurse. The median PD-scale score was 1 (IQR 0–1; *n* = 144) and the median CAP-D score was 4 (IQR 2–6). The Pearson coefficient of correlation between the sum scores of the PD-scale and the CAP-D was 0.89 (95% CI, 0.82 to 0.93; *p* < 0.001).

## Discussion

Delirium in critically ill children is a global problem and is increasingly recognized in this vulnerable patient group with a prevalence ranging from 4 up to 47% [4–10], and 10% in the current study. In this multicenter validation study, the PD-scale demonstrated high sensitivity (92%) and specificity (97%) for the detection of pediatric delirium – with one false-negative case and 14 false-positive cases. The likelihood ratios in this study can be labeled “strong” and provide robust evidence to rule in or rule out, respectively, the diagnosis of PD in most circumstances [30]. Further, the interrater reliability for the individual items had improved compared to our pilot study [21]. Altogether, these results confirm the measurement properties of the PD-scale established in our single-center pilot study [21]. Furthermore, the correlation between the PD-scale and the CAP-D was high, reflecting that both measure the same construct. Overall, the measurement properties of the PD-component of the SOS-PD scale are in line with those of the CAPD, pCAM-ICU, and psCAM-ICU [7–10, 31].

Schieveld and Zwieten recommended to develop a uniform observational screening tool across the entire age span [32] that could create a common diagnostic language and standardize the process of diagnosing delirium. However, developmental differences across the patient populations, such as in the elderly with dementia or comorbidity, with concomitant different expression patterns of delirium, make it unrealistic to develop a one-size-fits-all tool [33]. The primary goal is to implement delirium screening in daily practice, which currently is by far not the case [34]. Nurses are already expected to assess pain and level of sedation and adding a delirium screening tool increases the workload. However, not performing routine delirium screening might delay diagnosis and therapeutic interventions. The availability of different pediatric delirium screening tools will allow nurses and other healthcare professionals to choose a tool that best fits their needs, based on resources and ease of implementation. For instance, a screening tool that assesses the clinical picture “at the moment” such as the psCAM-ICU and the pCAM-ICU, or a tool such as the CAPD or the SOS-PD to assess the course during a nursing shift. Both the CAPD and the SOS-PD will detect delirium-associated fluctuations in brain dysfunction, such as consciousness and inattention, and therefore we believe the latter can be more appropriately used by caregiving nurses.

We propose that the effects of preventive and pharmacological and non-pharmacological treatment interventions be evaluated with the currently available validated delirium tools. Haloperidol is currently one of the most used antipsychotics for PD, although its efficacy for this indication is not proven [35–37]. There may be a larger role for nonpharmacological interventions than has been the case so far [38].

In this study, we found an overall estimated prevalence rate of delirium of 10% in critically ill children. This is lower than rates reported in recent studies, ranging from 17% up to 47% [8–10, 39]. The lower prevalence could be explained by the fact that our study patients were included 48 h after admission, in line with the Dutch guideline Pediatric Delirium [37], and therefore missing many self-limiting cases of emergence delirium. In this study, most of the delirious patients (75%) were diagnosed at day 5 or later. We may have missed patients with delirium; however, this study was not set up as an epidemiological study. Interestingly, although a single-center study on risk factors found that young age (≤2 years) is an independent risk factor of delirium, we identified the lowest estimated prevalence for this age group compared to older children in our multicenter study [39].

This multicenter validation study in four Dutch PICUs was performed in daily practice, in which the PD-scale assessments were made by well-trained PICU nurses. This increases the accuracy of the results of this study. Still, several limitations need to be addressed. First, the design of the study could have introduced observer bias in the “random sample”. To counteract this, we corrected for verification bias. Second, the study design may have introduced selection bias, because selected patients with a PD score <4 were chosen for psychiatric evaluation. Also, the paired observations, assessing the interrater reliability and construct validity, were performed in convenience samples and mostly in patients who were not delirious. The latter is a challenge in future studies. Third, the psychiatrist would know whether the consultation was for suspected delirium (SOS-PD score ≥4), as the attending physician customarily reported this 24 h/7 days. Besides, the psychiatric examination required additional information from the parents, attending physician, and attending nurse. These aspects could have introduced test bias. Fourth, the PD-scores were compared to the psychiatric examination according to the DSM-IV criteria, which was standardized, which is a strength. Yet we cannot rule out that the psychiatric teams in the participating hospitals assessed in different ways, with risk of subjectivity. Better accuracy could have been ensured by one team of child psychiatrists assessing all random samples, but this was impossible for logistical reasons. Fifth, even though the care-giving nurses were asked not to share the results of their PD-scale assessments with the psychiatrists, they could have non-verbally influenced the child psychiatrist.

## Conclusions

The SOS-PD scale has good interrater reliability and validity for screening by nurses of delirium in critically ill children. Early screening and routine monitoring of delirium with the SOS-PD scale could facilitate early capture and management of critically ill children with suspected delirium.

## Additional file

Additional file 1: **File S1.** Study procedure, flowchart study procedure. File S2 Interrater reliability (Cohen’s kappa) for the individual items of the PD-scale. (DOCX 77 kb)

## Abbreviations

CAPD-D: Cornell Assessment Pediatric Delirium
COSMIN: Consensus-based Standards for the Selection of Health Measurement Instruments
DSM: Diagnostic and statistical manual of mental disorders
ICC: Intraclass correlation coefficient
ICU: Intensive Care Unit
IWS: Iatrogenic withdrawal symptoms
pCAM-ICU: Pediatric Confusion Assessment Method for ICU patients
PD-scale: Pediatric Delirium Scale
PICU: Pediatric Intensive Care Unit
PRISM: Pediatric Risk of Mortality Score
psCAM-ICU: Preschool Confusion Assessment Method for ICU patients
RASS: Richmond Agitation Sedation Scale
SOS scale: Sophia Observation Withdrawal Symptoms Scale
SOS-PD scale: Sophia Observation Withdrawal Symptoms-Pediatric Delirium scale
