# Supplementary material for: Validation of the SOS-PD scale for assessment of pediatric delirium: a multicenter study
Source: Crit Care. 2018 Nov 20;22:309. doi: 10.1186/s13054-018-2238-z (PMC6247513; doi:10.1186/s13054-018-2238-z)
Supplement: Supplementary file 1 — File S1. Study procedure, flowchart study procedure. File S2 Interrater reliability (Cohen’s kappa) for the individual items of the PD-scale. (DOCX 77 kb) [file 13054_2018_2238_MOESM1_ESM.docx]

**Additional file 1:**

**S1 Study procedure**


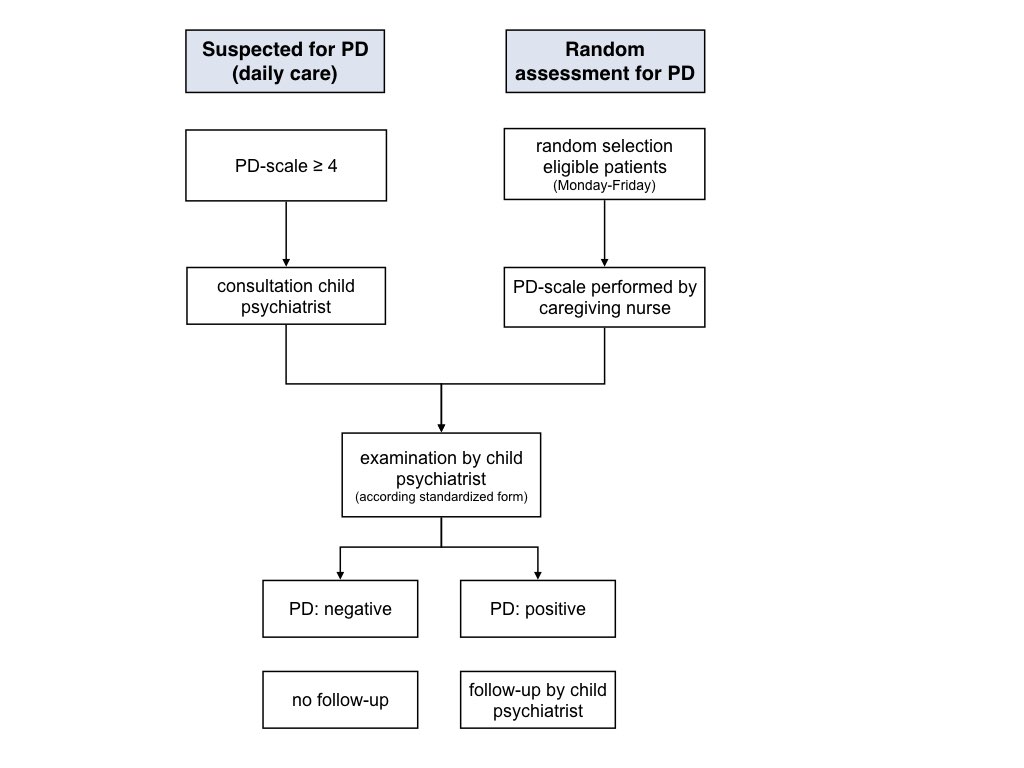


S2 Interrater reliability - items

| **Item** | **Kappa*** |
| --- | --- |
| Sweating | 0.913 |
| Agitation | 1.00 |
| Anxiety | 0.913 |
| Tremors | 0.848 |
| Motor disturbance | 1.00 |
| Muscle tension | 1.00 |
| Attentiveness | 1.00 |
| Purposeful acting | 1.00 |
| Lack of eye contact | 0.937 |
| Inconsolable crying | 1.00 |
| Grimacing | 1.00 |
| Sleeplessness | 1.00 |
| Hallucinations | 0.95 |
| Disorientation | 0.791 |
| Speech | 0.791 |
| Acute onset of symptoms | 1.00 |
| Fluctuation course | 1.00 |

* Cohen’s kappa
